# Effective Removal of Methylene Blue from Simulated Wastewater Using ZnO-Chitosan Nanocomposites: Optimization, Kinetics, and Isotherm Studies

**DOI:** 10.3390/molecules27154746

**Published:** 2022-07-25

**Authors:** Zakariyya Uba Zango, John Ojur Dennis, A. I. Aljameel, Fahad Usman, Mohammed Khalil Mohammed Ali, Bashir Abubakar Abdulkadir, Saja Algessair, Osamah A. Aldaghri, Khalid Hassan Ibnaouf

**Affiliations:** 1 Department of Chemistry, Al-Qalam University Katsina, Katsina 2137, PMB, Nigeria; 2 Department of Fundamental and Applied Science, Universiti Teknologi PETRONAS, Seri Iskandar 32610, Perak, Malaysia; jdennis100@gmail.com (J.O.D.); abubakar_g03619@utp.edu.my (B.A.A.); 3 Department of Physics, College of Science, Imam Mohammad Ibn Saud Islamic University (IMSIU), Riyadh 13318, Saudi Arabia; mkali@imamu.edu.sa (M.K.M.A.); saja.algessairs@imamu.edu.sa (S.A.); odaghri@imamu.edu.sa (O.A.A.); khiahmed@imamu.edu.sa (K.H.I.); 4 Department of Physics, Al-Qalam University Katsina, Katsina 2137, PMB, Nigeria; fahadusman@auk.edu.ng

**Keywords:** adsorption, chitosan, efficiency, kinetics isotherms, methylene blue, reusability

## Abstract

Successful synthesis of ZnO-chitosan nanocomposites was conducted for the removal of methylene blue from an aqueous medium. Remarkable performance of the nanocomposites was demonstrated for the effective uptake of the dye, thereby achieving 83.77, 93.78 and 97.93 mg g^−1^ for the chitosan, 5 wt.% ZnO-Chitosan and 10 wt.% ZnO-Chitosan, respectively. The corresponding adsorption efficiency was 88.77, 93.78 and 97.95 for the chitosan, 5 wt.% ZnO-Chitosan and 10 wt.% ZnO-Chitosan, respectively. Upon regeneration, good reusability of the nanocomposites was manifested for the continuous removal of the dye up to six consecutive cycles. The adsorption process was kinetically described by a pseudo-first order model, while the isotherms were best fitted by the Langmuir model.

## 1. Introduction

Chitosan is a chitin derivative obtained by treating crustacean shells with sodium hydroxide through the acetylation process [1]. Having the presence of three types of functional groups—amino/acetamido group, primary and secondary hydroxyl groups on its backbone—chitosan is widely used as modifying agents in polymer composites [2,3]. It has shown great potential in industrial applications due to its non-toxic nature, biodegradability, and biocompatibility [4,5]. It has affinity for various organic and inorganic materials due to its abundant reaction sites. It can adsorb and chelate metal ions [6], and interact with bioactive molecules [7]. Because of its porosity, biocompatibility, biodegradability, non-toxicity, and robustness, it has versatile application in the preparations of novel chitosan-based materials for various applications such as pharmaceutical, nutraceutical [8], biomedical [9,10], fertilizer delivery [11], CO_2_ capture [5], catalysis [12] and wastewater remediations [13,14].

However, chitosan in its pure form is not as effective as its modified form. It is plagued by solubility deficiency, especially under acidic environments, which affects its mechanical properties for water related applications [15,16]. Thus, surface modification has been put forward to improve its structural properties. To increase the number of exposed active sites, various chemical or physical modifications have been adopted. Carbone nanotubes (CNTs) have been employed for the enhancement of the thermal stability of chitosan membranes and its reinforcement [17,18]. The use of the clay minerals such as smectite and sepiolite has been shown to improve its mechanical properties and resistance to aqueous media [19]. Similarly, the composites of chitosan with alginate have shown good chemical, thermal and moisture resistance [4]. Chitosan/halloysite composite membranes with excellent physico-chemical and thermal properties were also discovered [20]. Surfactant-modified chitosan (SMCS) beads have recently been reported for effective adsorption of heavy metals from the aqueous medium [21].

Recently, surface coating of the chitosan with metal oxide nanoparticles has gained the recognition of researchers for adsorption, catalysis, degradation, and biomedical applications [22,23]. A facile and greener synthesis of chitosan-FeO nanocomposite was reported by Bharathi et al. (2019). The good structural and absorption properties of the biopolymer material were ascertained, and its potential antibacterial activity was evaluated [9]. Neeraj et al. (2016) investigated the elimination of arsenic from the aqueous solution using a nanocomposite of chitosan coated with iron-oxide. The porosity of the material was confirmed with a cumulative pore volume of 0.0362 m^3^/g and pore diameter of 32.46 nm. Thus, the maximum monolayer adsorption capacity of the material was 267.2 mg/g [24]. Synthesis and characterizations of nano hydrogel beads of chitosan/agar/SiO_2_ composites were also reported for effective pharmaceutical adsorption from environmental waters, achieving over 99% adsorption of naproxen within 15 min of the batch adsorption process [25].

Rapid detection of dyes in various water bodies has been an environmental phenomenon over the years [26,27]. In this research, we aimed at synthesizing water resistant ZnO-chitosan nanocomposites for the efficient adsorption of dyes from the aqueous medium. Methylene blue was chosen as model dye due to its large consumption by textile, leather, tannery, paper, and pulp processing industries [28]. Thus, the adsorption parameters, kinetics, isotherms, regeneration, and reusability of the adsorbent materials were evaluated for optimum uptake of the dye from the aqueous medium.

## 2. Results

### 2.1. Materials

Chitosan (85% deacetylated, Molecular weight of 400,000 Da) is purchased from Golden-Shell Biochemical (Yuhuan, Zhejiang, China). The crystal violet (Molecular Formula C_25_N_3_H_30_Cl, MW 407.979 g/mol and Density: 1.19 g/cm^3^) and the zinc oxide nano powder of particle size 50–100 nm and 98% purity were purchased from Sigma-Aldrich (St. Louis, MO, USA). Acetic acid and ethanol were purchased from Avantis Laboratory, Perak, Malaysia. All other chemicals not mentioned here were of high purity and of analytical reagent (AR) grade and were used as received. Double distilled water was used throughout the study.

### 2.2. Synthesis of ZnO-Chitosan Nanocomposite

The nanocomposites were synthesized by dissolving 10 mg of ZnO Nano powder in 90 mL ethanol with constant stirring for 15 min. A 50 mg of Chitosan was dispersed in 20 mL of 0.1 M acetic acid and stirred for 15 min. The 10 mL ZnO solution was gradually added to the dispersed chitosan. The mixture was continuously stirred for 2 h, and the resulting mixture was centrifuged at 4000 rpm, filtered and washed thoroughly with ethanol and double distilled water. The obtained residue was dried overnight in an oven at 100 °C and stored in vacuum. It was labeled as 10 wt.% ZnO-chitosan. For the preparation of 5 wt.% ZnO-chitosan, the same method was employed; only 5 mL of the ZnO solution was used.

### 2.3. Characterizations

The materials were examined by Scanning Electron Microscopy (SEM) (SU8020, Hitachi, Tokyo, Japan) for the morphology analysis. The samples were coated with gold by a Polaran (SC 515) sputter coater to make it electrically conductive. N_2_ adsorption–desorption of the samples were determined by a TriStar II (3020) Micromeritics porosity analyzer, Norcross, GA, USA. The sample was heated at 60 °C, and the analysis was conducted under a nitrogen stream with adsorption–desorption isotherms at 77 K.

### 2.4. Preparation of Methylene Blue Solution

The dye stock solution was prepared by dissolving 1000 mg of the methylene blue in 100 mL distilled water. The solution was transferred to a 1000 mL volumetric flask, and distilled water was added to the mark, making a concentration of 1000 mg L^−1^, which was kept in a refrigerator prior to the adsorption experiments. The solutions of different concentrations used in various experiments were obtained by a dilution of the stock solutions.

### 2.5. Batch Adsorption Experiment

The adsorption behavior of chitosan and the nanocomposites were evaluated for the methylene blue adsorption. Batch experiments were conducted using 50 mL of 100 mg L^−1^ of the dye solutions in 100 mL conical flasks with the adsorbent of 6 g/L. The conical flasks were placed in a thermostatic shaker at 250 rpm and 30 °C. The absorbance of the solutions was analyzed by using a UV–vis spectrophotometer (Varian CARY 50 probe) at 665 nm.

The optimum time can be obtained from the plot of adsorption capacity versus time using the formula:

(1) $$q_{t}=\frac{(C_{o}-C_{t})V}{w}$$

In addition, the equilibrium time for the adsorption of the dye is obtained using the formula:

(2) $$q_{e}=\frac{(C_{o}-C_{e})V}{w}$$

and the removal efficiency (%R) is calculated from the formula:

(3) $$\left(\%R\right)=\frac{C_{o}-C_{e}}{C_{o}}\times 100$$

where $C_{o}$ and $C_{e}$ are initial and equilibrium concentrations of the dye (mg L^−1^), respectively.

### 2.6. Adsorption Kinetics

The data for the batch adsorption studied were evaluated by pseudo-first order, pseudo-second order, and intra-particle diffusion described by the Equations (5)–(7), respectively, for the determination of the best kinetic model:

(4) $$\ln\left(q_{e}-q_{t}\right)=\ln q_{e}-k_{1}t$$

(5) t/qt=1k2qe2+tqe

(6) $$q_{t}=k_{p}t^{1/2}+C$$

where $q_{e}$ and $q_{t}$ represent the amounts of dye adsorbed (mg/g) at equilibrium and time *t*, respectively. $k_{1}$ (1/min) and $k_{2}$ (g mg^−1^ min^−1^) are the rate constants of the pseudo first-order and pseudo second-order adsorption kinetic model, respectively. $k_{p}$ is the intraparticle diffusion rate constant and *C* being constant.

### 2.7. Adsorption Isotherms

To evaluate the isotherms fitting on the adsorption data, the models of Langmuir, Freundlich, and Temkin were employed as represented by Equations (7)–(9), respectively:

(7) $$\frac{C_{e}}{q_{e}}=\frac{1}{K_{L}q_{m}}+\frac{1}{q_{m}}C_{e}$$

(8) $$\log q_{e}=\log K_{F}+\frac{1}{n}\log C_{e}$$

(9) $$q_{e}=\frac{RT}{b_{T}}\ln A_{T}+\left(\frac{RT}{b_{T}}\right)\ln C_{e}$$

where $q_{m}$ (mg g^−1^) is the Langmuir adsorption capacity, $K_{L}$ is the Langmuir constant (L mg^−1^), $K_{F}$ is the Freundlich constant (L mg^−1^), 1/*n* represents the adsorption intensity, $b_{T}$ (kJ mol^−1^) represents the Temkin constant which relates to the heat of adsorption, and $A_{T}$ is the equilibrium binding constant corresponding to the maximum binding energy (L g^−1^). *T* is the absolute temperature (K), and *R* (8.314 J mol^−1^ K^−1^) is the Universal gas constant.

The favorability of the adsorption process was determined from the Langmuir constant given by Equation (10):

(10) $$R_{L}=\frac{1}{1+C_{0}K_{L}}$$

When the value of $R_{L}$ is less than unity, the adsorption is favorable and when greater than unity is considered as unfavorable. In addition, when $R_{L}$ is 0, the adsorption is irreversible and linear when it is unity.

Moreover, these models were statistically analyzed using regression to determine the coefficient of determination (*R*^2^), root mean square error (*RMSE*), and Akaike information criterion (*AIC*) to assess the model performance, using Equations (11)–(13):

(11) $$R^{2}=1\frac{\sum {\left(q_{e\;exp}-q_{e\;cal}\right)}^{2}}{\sum {\left(q_{e\;exp}\right)}^{2}}$$

(12) $$RMSE=\sqrt{\sum_{n=1}^{i}{\left(q_{e\;exp}-q_{e\;model}\right)}^{2}}$$

(13) $$AIC=n\ln\left(\frac{SSE}{n}\right)+2n_{p}+\frac{2n_{p}\left(n_{p}+1\right)}{n\left(n_{p}+1\right)}$$

where $q_{e\;exp}$ and $q_{e\;model}$ represent experimental and model adsorption capacity, *n* is the number of observations, and *p* denotes the number of parameters. *SSE* is the sum of the square errors obtained. Higher *R*^2^ value indicates better linearity of the models while smaller *RMSE* and *AIC* indicate better fitting of the model.

### 2.8. Effect of Adsorbent Dosage

The adsorbents dosage was varied from 1–6 g L^−1^, and, using the initial dye concentration of 100 mg L^−1^ and pH at 4, the adsorption experiment was conducted. The removal efficiency (%R) was plotted against adsorbent dosage

### 2.9. Effect of pH

Solution pH affects the adsorption process by affecting both aqueous chemistry and surface binding sites of the adsorbent. In this work, the pH range was studied from 2–12 using an initial dye concentration of 100 mg L^−1^. The pH was adjusted with 0.1 M HCl or NaOH and measured with pH-meter model HI 8014, Hanna Instruments (Padua, Italy).

### 2.10. Adsorbent Regeneration and Reusability Test

For the adsorbent regeneration and reusability, an adsorption experiment was conducted with an initial dye concentration of 100 mg L^−1^ and adsorbent dosage of 6 g L^−1^ at room temperature and stirring rate of 250 rpm. After the adsorption, the adsorbent was centrifuged, filtered, and washed with 0.1 M NaOH solution and then several times with distilled water to remove all the traces of the dye [29,30]. The experiments were repeated several times using the same procedure.

## 3. Discussion

### 3.1. Characterizations

**Figure 1:**
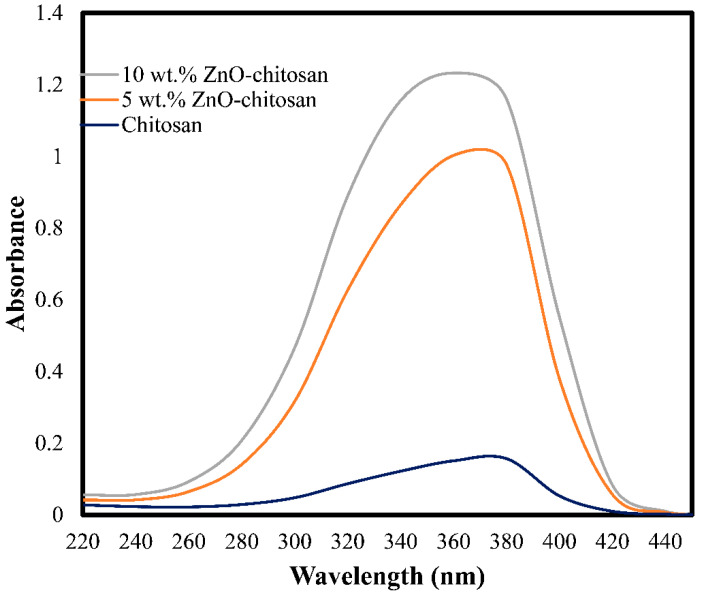
UV-visible absorbance spectra of 5 wt.% ZnO-chitosan and 10 wt.% ZnO-chitosan nanocomposites.

The characteristic absorption band of the pristine chitosan and nanocomposites was studied using UV-Visible absorption spectroscopy. As shown in Figure 1, the composites have exhibited strong absorption band at around 350–380 nm, compared to the pristine chitosan with the corresponding calculated band gap of 3.62 eV. This corresponds to the band gap of the pristine ZnO nanoparticles having a value of 3.37 eV with an absorption maximum of 368 nm as previously reported by Rao et al. [31]. Thus, the spectra confirmed the dispersion of the ZnO nanoparticles on the surface of the chitosan and the good adsorption properties of the ZnO-chitosan nanocomposites.

**Figure 2:**
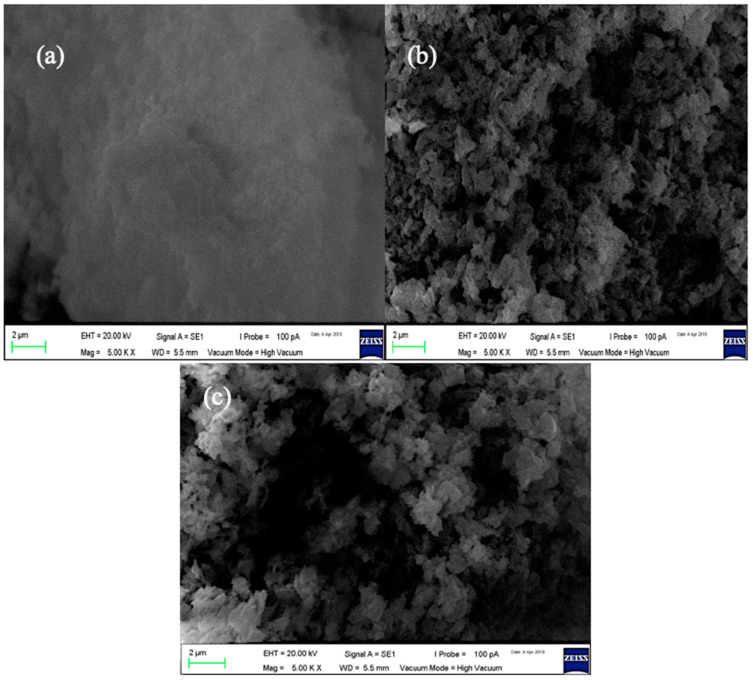
SEM of Chitosan (**a**) 5 wt.% ZnO-chitosan (**b**) 10 wt.% ZnO-chitosan (**c**) at 1 K and 5 K magnifications, respectively.

**Figure 3:**
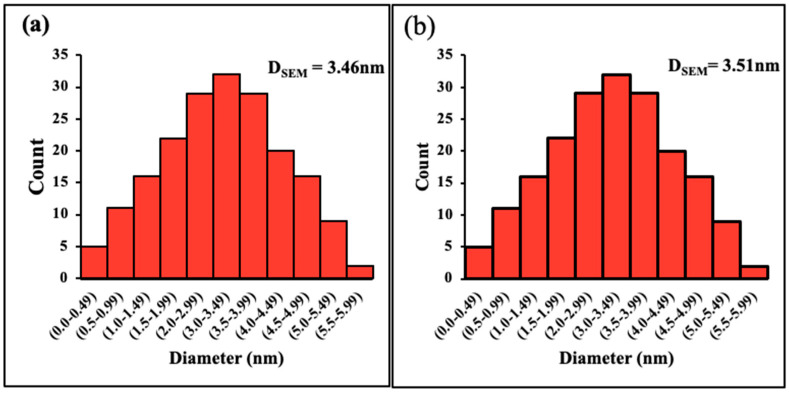
Particle size distribution of ZnO in 5 wt.% ZnO-chitosan (**a**) and 10 wt.% ZnO-chitosan (**b**) nanocomposites.

The morphology of the prepared composites is shown the SEM images as depicted in Figure 2. It indicated the spherical or elliptical shape of the ZnO nanoparticles on the surface of the chitosan. Previous studies have indicated that the presence of metal nanoparticles enhanced the adsorption performance of the pristine chitosan, such as the work of Rahmi et al. (2019) for the adsorption of Hg (II) and Cd (II) onto highly crystalline Fe_2_O_3_@chitosan nanocomposite [16]. Most recent is the finding of Moradi et al. for the naproxen adsorption onto Chitosan/agar/SiO_2_ nano hydrogels [25]. The average particle size was 3.46 and 3.51 nm for the 5 wt.% and 10 wt.% ZnO-chitosan, respectively, as depicted in Figure 3.

The porosity of the adsorbent materials is vital for adsorption studies as the pores in the adsorbents offer more adsorption sites for the guest molecules. Adsorbents having higher N_2_ adsorption–desorption isotherms often presented higher adsorption sites for the host molecules. The specific Brunauer–Emmette–Teller (BET) surface area of the pristine chitosan was 4.22 m^2^ g. However, for the nano composites, BET surface area of 45.70 and 49.21 m^2^ g^−1^ were recorded for 5 wt.% ZnO-chitosan and 10 wt.% ZnO-chitosan samples, respectively, as shown in Table 1. The corresponding values for pore volumes and the average pore diameter according to the Barrett–Joyner–Halenda (BJH) were highlighted in Table 1. The higher surface area of the nanocomposites revealed their good efficiency for the uptake of the dye molecules. Previous reports have also indicated that the synergetic effect of metal-oxide nanoparticles affects the overall surface area of pristine chitosan [23].

### 3.2. Effect of Contact Time

**Figure 4:**
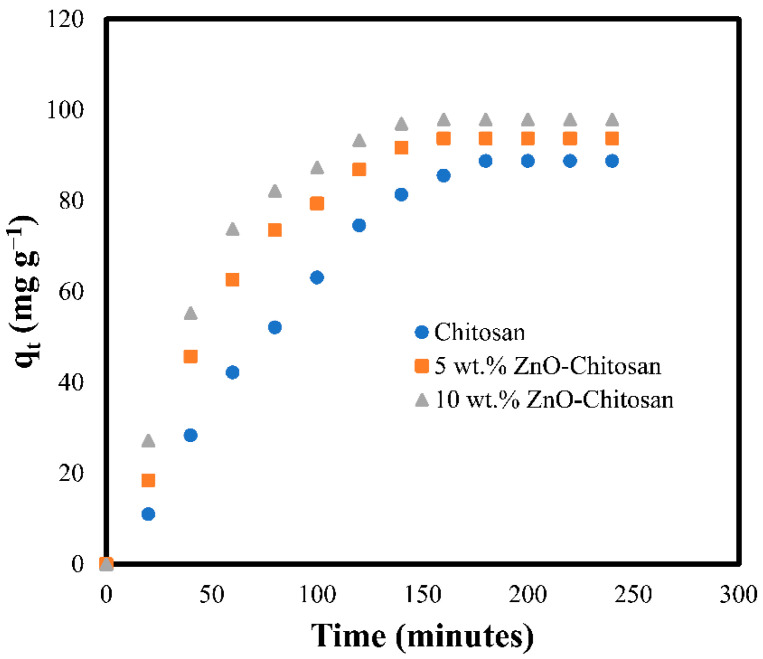
Effect of contact time for the dye adsorption onto chitosan and the nanocomposites.

Contact time is paramount variable in adsorption processes. Thus, the effect of contact time on the dye adsorption onto the pristine chitosan and the nanocomposites were investigated. Figure 4 has shown the increase in adsorption capacity ($q_{t}$ mg g^−1^) with the contact time for both chitosan and nanocomposites. This might be attributed to the diffusion of dye molecules from the surface of the solution onto the surface of the adsorbents [32]. However, with time, the adsorption capacity becomes moderate, probably due to the migration of dye molecules to inner pores of the adsorbents. The equilibrium was achieved at 160 min with equilibrium adsorption capacity of 93.78 mg g^−1^ and 97.93 mg g^−1^ for 5 wt.% ZnO-Chitosan and 10 wt.% ZnO-Chitosan, respectively. In comparison, the pristine chitosan has a $q_{e}$ value of 83.77 mg g^−1^. These results indicated an improvement in the adsorption capacity of the nanocomposites when compared with the chitosan. A similar observation was reported when the chitosan was modified with PVA/TiO_2_ [33].

### 3.3. Adsorbent Dosage

**Figure 5:**
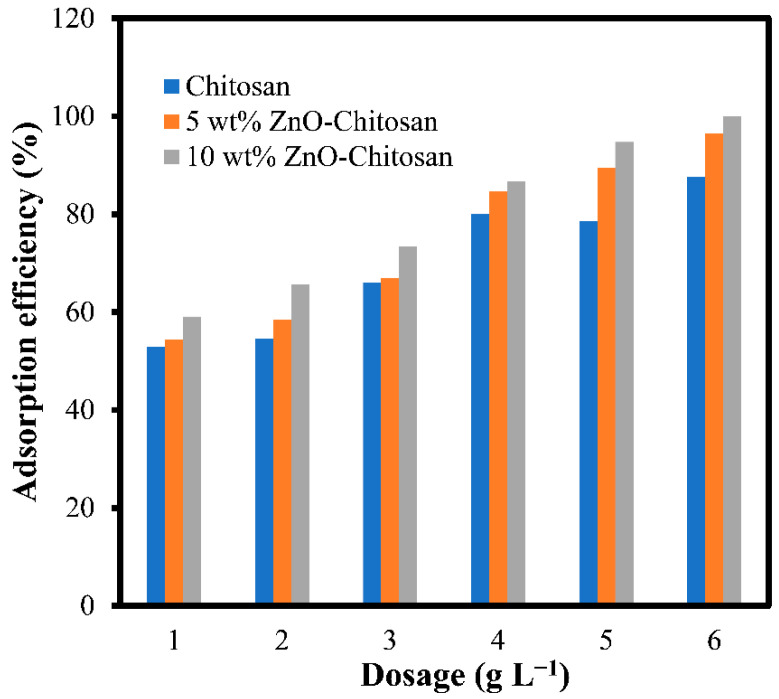
Effects of adsorbent dosage for the dye adsorption onto the chitosan and the nanocomposite.

A promising adsorbent material must be able to remove considerable amounts of adsorbate at low doses. This feature is paramount to reduce operational costs and minimize the risks of secondary pollution [34,35]. From the plot of Figure 5, the removal efficiency (%R) against adsorbent dosage (g L^−1^), it was shown that adsorption capacity increased with the amount of the adsorbent. This was due to the increase in number of active sites. Thus, adsorbent dosage of 6 g L^−1^ is required to remove the dye concentration of 100 mg L^−1^, achieving the higher adsorption efficiency of 87.54, 96.39, and 99.95% for the chitosan, 5 wt.% ZnO-chitosan, and 10 wt.% ZnO-chitosan composites, respectively [36,37].

### 3.4. Effect of Dye Concentration

**Figure 6:**
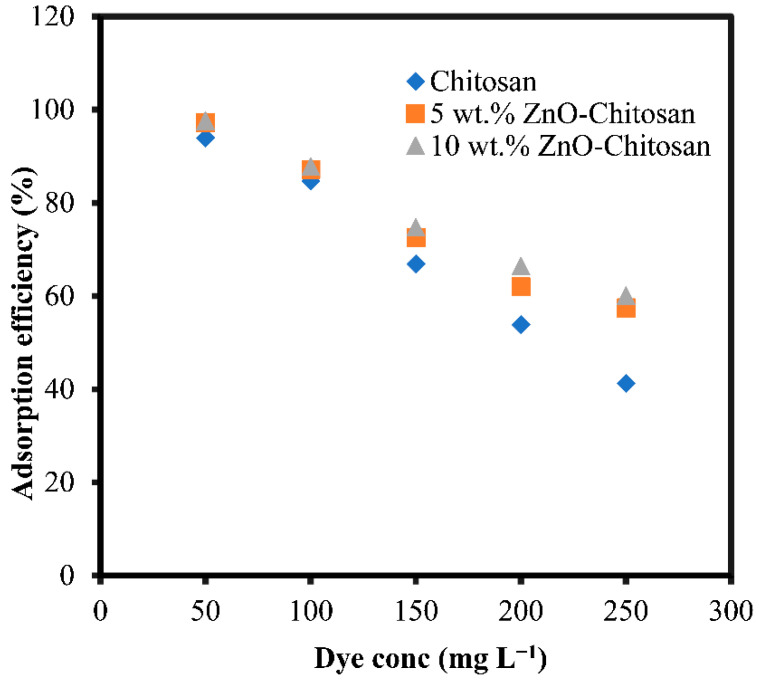
Effect of initial dye concentrations for the dye adsorption onto the chitosan and nanocomposites.

The adsorption of the dye onto adsorbents was studied at different initial concentrations. From Figure 6, the adsorption efficiency decreased for all the adsorbents when the initial concentration was changed from 50 to 250 mg L^−1^. For the pristine chitosan, the adsorption efficiency drastically decreased from 96.17 to 42.21%, while, for the nanocomposites, the adsorption efficiency decreased from 99.53 to 58. 83% and from 99.95 to 61.51% for the 5 wt.% ZnO-chitosan and 10 wt.% ZnO-chitosan, respectively. The decrease in the adsorption efficiency at higher concentration was due to the limited number of the adsorption sites available for the uptake of the dye. As the adsorption sites became saturated, no more adsorption occurred. However, the adsorption capacity ($q_{e}$ mg g^−1^) increased as the initial concentration of the dye was increased due to the mass driving force that enables the transfer of the dye molecules to the active sites of the adsorbents.

### 3.5. Influence of pH on Adsorption

**Figure 7:**
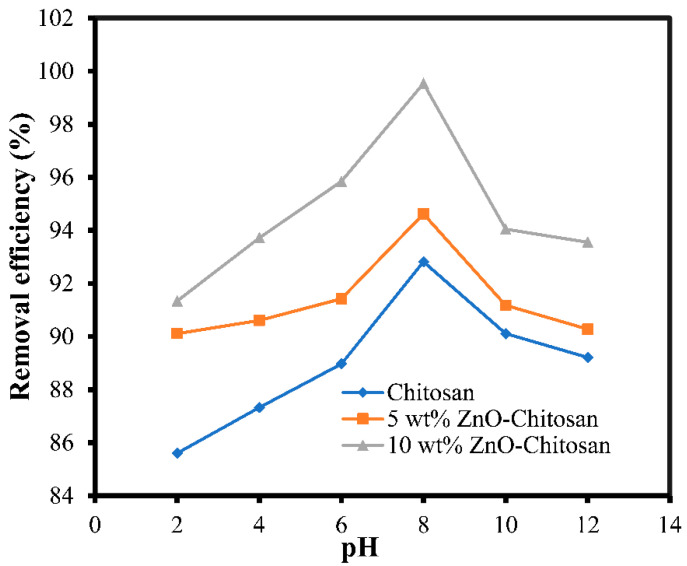
Effects of pH for the dye adsorption onto the chitosan and the nanocomposites.

The adsorption capacity gradually increased at the acidic pH until it reached the peak at the pH of 8. It then started to decline at the alkaline pH (Figure 7). The increase in the adsorption capacity observed at the lower pH was resulted from the attraction of the amino group on the surface of the chitosan for the hydroxonium ion on the surface of the solution [38]. Also at the basic pH above 7, deprotonation of the active sites of the surface of the adsorbent occurred, thus repulsed with the OH^−^ on the surface of the [39,40]. The presence of excess H^+^ and OH^−^ in the acidic or basic solution competes with the dye for the adsorption sites, which resulted in lower adsorption capacity.

### 3.6. Kinetics of Adsorption

The mechanism and the rate controlling step of the adsorption process was evaluated by the kinetics models of pseudo-first order and pseudo-second order intra-particle diffusion. Among models, the pseudo-first order has shown the best calculated adsorption capacities of 85.308, 98.397 and 103.048 mg g^−1^ for the chitosan, 5 wt.% ZnO-chitosan and 10 wt.% ZnO-chitosan, respectively. This has been in good agreement with the experimental results. Additionally, the fitting data of the model have shown best *R*^2^ values 0.998, 0.995 and 0.959 for the chitosan, 5 wt.% ZnO-chitosan and 10 wt.% ZnO-chitosan, respectively. Similarly, the statistical values for the linear regression analysis of MSE, RMSE and AIC of the model were also in good agreement with the finding as highlighted in Table 2. Thus, the adsorption of the dye onto the pristine chitosan and the nanocomposites best described the abundant adsorption sites as depicted by the improvement in the BET surface area upon the dispersion of the ZnO nanoparticle on the surface of the chitosan.

### 3.7. Isotherms of Adsorption

The isotherms studies were used to describe the interaction between the dye and the adsorbents when the equilibrium is attained. Of the models studied (Table 3), Langmuir fitting was the most consistent for the adsorption data according to the obtained *R*^2^ values and the statistical regression analysis as highlighted in Table 4. The Langmuir adsorption capacity ($q_{m}$) was 68.077, 87.471 and 90.976 mg/g for the chitosan, 5 wt.% ZnO-Chitosan and 10 wt.% ZnO-chitosan, respectively, indicating the monolayer formation and the good adsorption capacity of the adsorbents [27]. Similarly, the corresponding *R_L_* values were 0.043, 0.224 and 0.019 for the chitosan, 5 wt.% ZnO-Chitosan and 10 wt.% ZnO-chitosan, respectively, signifying the favorability of the adsorption. Thus, the overall adsorption process is said to occur via monolayer formation. Previously, Zhang et al. have reported similar observations for the adsorption of cesium chitosan-vermiculite composite [41].

### 3.8. Adsorbent Regeneration and Reusability

**Figure 8:**
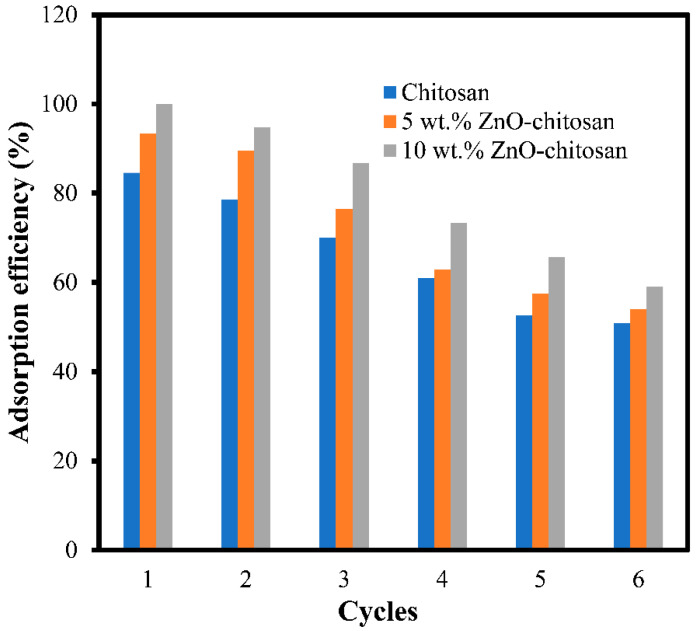
Reusability of chitosan and composites for the dye adsorption.

Results from regeneration and reusability studies indicated that both chitosan and the nanocomposites can withstand the dye adsorption for number of repeated usages with good efficiency. From Figure 8, the adsorption efficiency of the chitosan dropped from 84.54% to 50.93%, whereas it dropped from 93.39% to 54.04% and 99.95% to 59.05% for the 5 wt.% ZnO-chitosan and 10 wt.% ZnO-chitosan, for the 1st and 6th cycles, respectively. This reaffirmed that both chitosan and composites are good adsorbents for the dye removal over a repeated number of adsorption cycles.

Literature studies have also restated the good performance of the nanocomposites in comparison to other chitosan and composites reported. The efficiency of the ZnO-chitosan nanocomposites could be deduced from the higher adsorption capacities of the materials and the shorter equilibration time than most of the adsorbents previously employed. Thus, the relevance of this work in the field of pollutants’ remediation from environmental waters.

## 4. Conclusions

ZnO nanoparticles were successfully dispersed on chitosan, forming 5 wt.% ZnO-Chitosan and 10 wt./% ZnO-Chitosan nanocomposites. The UV-visible analysis has shown the good adsorption of the composites, while the SEM analysis described the surface morphology of the nanocomposites with the spherical or elliptical shape of the ZnO nanoparticles, indicating the formation of the ZnO-chitosan nanocomposites. The N_2_ adsorption–desorption analysis revealed the good porosities of the composites for the uptake of the guest molecules. The adsorption studies for the removal of methylene blue from the aqueous medium demonstrated efficiency of the nanocomposites and its higher adsorption capacity compared to the pristine chitosan with the equilibrium attained within 160 min. The adsorption capacities were 83.77, 93.78 and 97.93 mg g^−1^ for the chitosan, 5 wt.% ZnO-Chitosan and 10 wt.% ZnO-Chitosan, respectively. The good adsorbent properties of the materials were demonstrated for the efficient adsorption of the methylene blue up to six cycles, achieving remarkable adsorption efficiency. The kinetics and isotherms were governed by pseudo-first order and Langmuir model, respectively. Thus, the nanocomposites can be employed as good adsorbents for pollutants removal from the environmental waters. However, for real sample application, the multi-component adsorption system should be employed using column technology and a suitable experimental design model.

## Figures and Tables

**Table 1:** N_2_ adsorption–desorption properties of chitosan and the composites.

Properties	Chitosan	5 wt.% ZnO-Chitosan	10 wt.% ZnO-Chitosan
BET surface area (m^2^ g^−1^)	4.22	45.70	49.21
Pore volume (m^3^ g^−1^)	0.013	0.025	0.025
Pore sizes (nm)	4.15	4.62	4.62

**Table 2:** Kinetics models for the dye adsorption onto the chitosan and the nanocomposites.

Model	Chitosan	5 wt.% ZnO-Chitosan	10 wt.% ZnO-Chitosan
***q_e_*** (Experimental mg g^−1^)	88.771	93.780	97.951
**Pseudo-first order**			
***q_e_*** (Calculated mg g^−1^)	85.308	98.397	103.048
$K_{1}$ (min^−1^)	0.010	0.012	0.013
*R* ^2^	0.998	0.995	0.959
*R*^2^ adj	0.978	0.983	0.943
*MSE*	0.020	0.027	0.026
*RMSE*	0.141	0.163	0.160
*AIC*	−33.141	−30.904	−31.197
**Pseudo-second order**			
***q_e_*** (Calculated mg g^−1^)	16.250	27.651	30.150
$K_{2}$ (g mg^−1^ min^−1^)	0.000	0.000	0.000
*R* ^2^	0.707	0.772	0.909
*R*^2^ adj	0.665	0.739	0.896
*MSE*	0.172	0.063	0.024
*RMSE*	0.414	0.251	0.156
*AIC*	−14.119	−23.125	−31.299
**Intra-particle diffusion**			
*K_p_*	0.763	0.702	0.673
*C*	15.275	29.205	36.997
*R* ^2^	0.979	0.897	0.842
*R*^2^ adj	0.976	0.882	0.820
*MSE*	22.302	131.873	212.649
*RMSE*	4.723	11.484	14.493
*AIC*	29.680	46.067	49.975

**Table 3:** Isotherms studies for the dye adsorption onto chitosan and composites.

	Chitosan	5 wt.% ZnO-Chitosan	10 wt.% ZnO-Chitosan
**Langmuir**			
*Q_m_* (mg g^−1^)	68.077	87.471	90.976
*K_L_* (L mg^−1^)	0.222	0.437	0.524
*R_L_*	0.043	0.224	0.019
*R* ^2^	0.976	0.982	0.983
*R*^2^ adj	0.969	0.977	0.977
*MSE*	0.005	0.002	0.001
*RMSE*	0.075	0.045	0.039
*AIC*	−24.881	−29.558	−31.091
**Freundlich**			
*K_F_* (L g^−1^)	13.426	12.368	12.075
*n_F_*	4.132	9.699	17.241
*R* ^2^	0.868	0.749	0.667
*R*^2^ adj	0.824	0.661	0.556
*MSE*	0.014	0.017	0.017
*RMSE*	0.117	0.129	0.132
*AIC*	−20.049	−19.807	−18.799
**Temkin**			
*b_T_* (kJ mol^−1^)	216.024	205.905	195.334
*A_T_* (L g^−1^)	7.745	5.039	5.419
*R* ^2^	0.911	0.795	0.718
*R*^2^ adj	0.882	0.726	0.624
*MSE*	43.845	76.863	88.748
*RMSE*	6.617	8.767	9.421
*AIC*	20.349	23.156	23.875

**Table 4:** Comparison of adsorption of various dyes onto chitosan and modified chitosan.

Adsorbent	Dye	Conc (mg L^−1^)	$q_{e}\;(\mathrm{mg}\;g^{-1})$	Equilibrium Time	Ref
Chitosan	Reactive yellow Reactive black	300	- -	800 min	[42]
Chitosan	Methylene blue	10	-	4 h	[43]
Chitosan	Methylene blue	10	9.88	30 min	[44]
Chitosan	Acid dye Basic dye Direct dye Reactive dye	100	58.50 7.30 52.30 50.40	5 h	[45]
Chitosan-cyclodextrin	Direct blue 78	300	10.80	350 min	[46]
NCCA NCCF	Red 60	100	5.86 3.40	240 min	[47]
Chitosan@Fe_3_O_4_	Congo red	100	56.66	600 min	[48]
Chitosan/Al_2_O_3_/magnetite	Methyl orange	20	47.60	40 min	[49]
Sr_3_._8_Fe_25_._7_O_70_._4_-chitosan	Crystal violet Basic red	50	29.46 32.16	30 min	[50]
Chitosan	Methylene blue	100	88.77	160 min	This work
5 wt.% ZnO-Chitosan	Methylene blue	1	93.78	160 min	This work
10 wt.% ZnO-Chitosan	Methylene blue	100	97.93	160 min	This work

Chitosan-4-nitroacetophenone/CuO-CeO_2_-Al_2_O_3_ (NCCA); Chitosan-4-nitroacetophenon/CuO-CeO_2_-Fe_2_O_3_ (NCCF).

## Data Availability

The data presented in this study are available in Supplementary Material.

## Supplementary Materials

The following supporting information can be downloaded at: https://www.mdpi.com/article/10.3390/molecules27154746/s1, Figure S1: Pseudo-first order model plot for methylene blue adsorption onto chitosan and the nanocomposites; Figure S2: Pseudo-second order model plot for methylene blue adsorption onto chitosan and the nanocomposites; Figure S3: Intraparticle adsorption model plot for methylene blue adsorption onto chitosan and the nanocomposites; Figure S4: Langmuir isotherm model plot for methylene blue adsorption onto chitosan and the nanocomposites; Figure S5: Freundlich isotherm model plot for methylene blue adsorption onto chitosan and the nanocomposites; Figure S6: Temkin isotherm model plot for methylene blue adsorption onto chitosan and the nanocomposites.

## Sample Availability

Samples of the compounds are not available from the authors.
