# Supplementary material for: Effective Removal of Methylene Blue from Simulated Wastewater Using ZnO-Chitosan Nanocomposites: Optimization, Kinetics, and Isotherm Studies
Source: Molecules. 2022 Jul 25;27(15):4746. doi: 10.3390/molecules27154746 (PMC9332308; doi:10.3390/molecules27154746)
Supplement: Supplementary file 1 [file molecules-27-04746-s001.zip › molecules-1806797-supplementary.pdf]

Effective Removal of Methylene Blue from Simulated Wastewater Using ZnO-Chitosan Nanocomposites: Optimization, Kinetics, and Isotherm Studies

Zakariyya Uba Zango [1,*], John Ojur Dennis [2], A.I. Aljameel [3,*], Fahad Usman [4], Mohammed Khalil Mohammed Ali [3], Bashir Abubakar Abdulkadir [2], Saja Algessair [3], Osamah A. Aldaghri [3] and Khalid Hassan Ibnaouf [3]

[1] Department of Chemistry, Al-Qalam University Katsina, PMB 2137, Katsina, Nigeria
[2] Department of Fundamental and Applied Science, Universiti Teknologi PETRONAS, Seri Iskandar 32610, Perak, Malaysia; jdennis100@gmail.com (J.O.D.); abubakar_g03619@utp.edu.my (B.A.A.)
[3] Department of Physics, College of Science, Imam Mohammad Ibn Saud Islamic University (IMSIU), Riyadh 13318, Saudi Arabia; mkali@imamu.edu.sa (M.K.M.A.); saja.algessairs@imamu.edu.sa (S.A.); odaghri@imamu.edu.sa (O.A.); khiahmed@imamu.edu.sa (K.H.I.)
[4] Department of Physics, Al-Qalam University Katsina, PMB 2137, Katsina, Nigeria; fahadusman@auk.edu.ng (F.U.)
* Correspondence: zakariyyazang@auk.edu.ng (Z.U.Z.); aialjameel@imamu.edu.sa (A.I.A.)

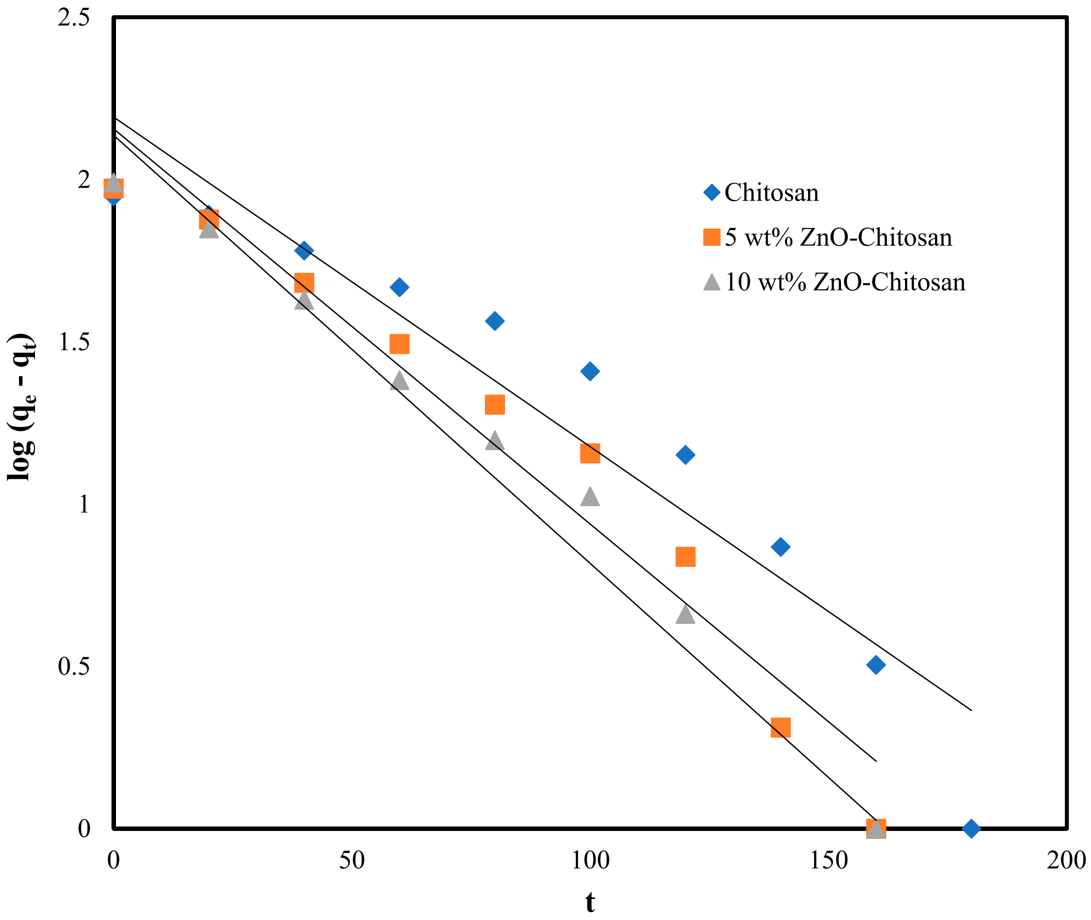

Figure S1. Pseudo-first order model plot for methylene blue adsorption onto chitosan and the nanocomposites.

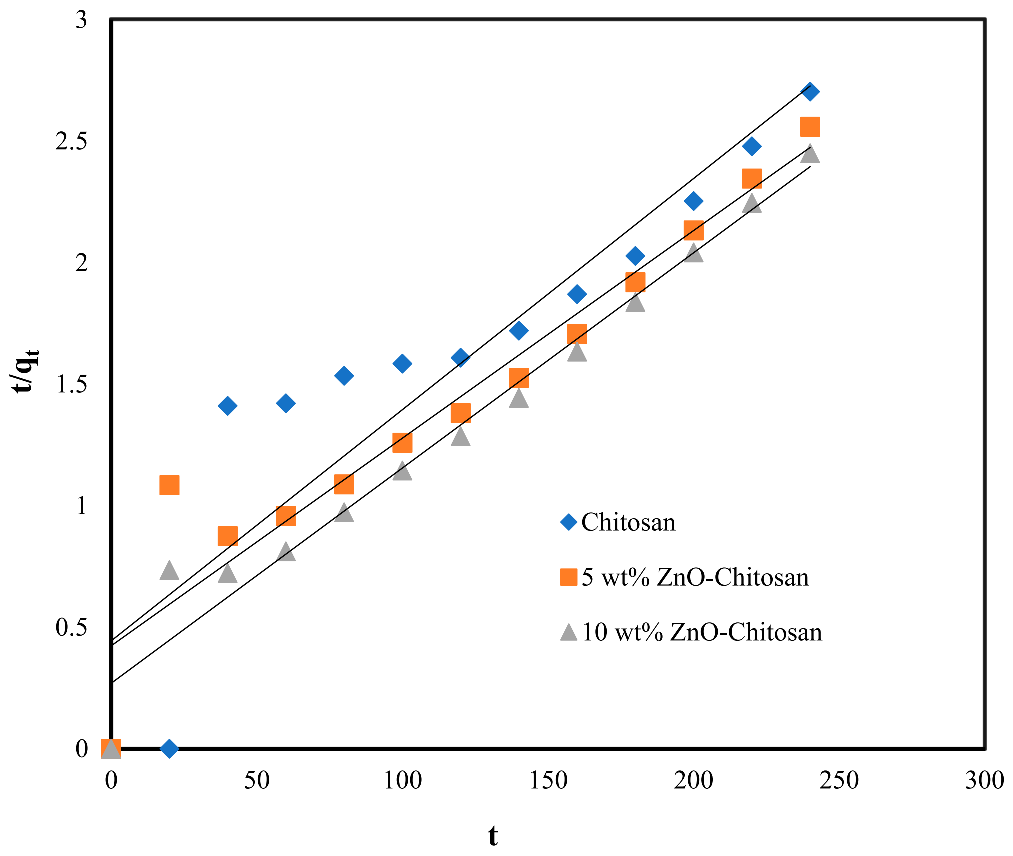

Figure S2. Pseudo-second order model plot for methylene blue adsorption onto chitosan and the nanocomposites.

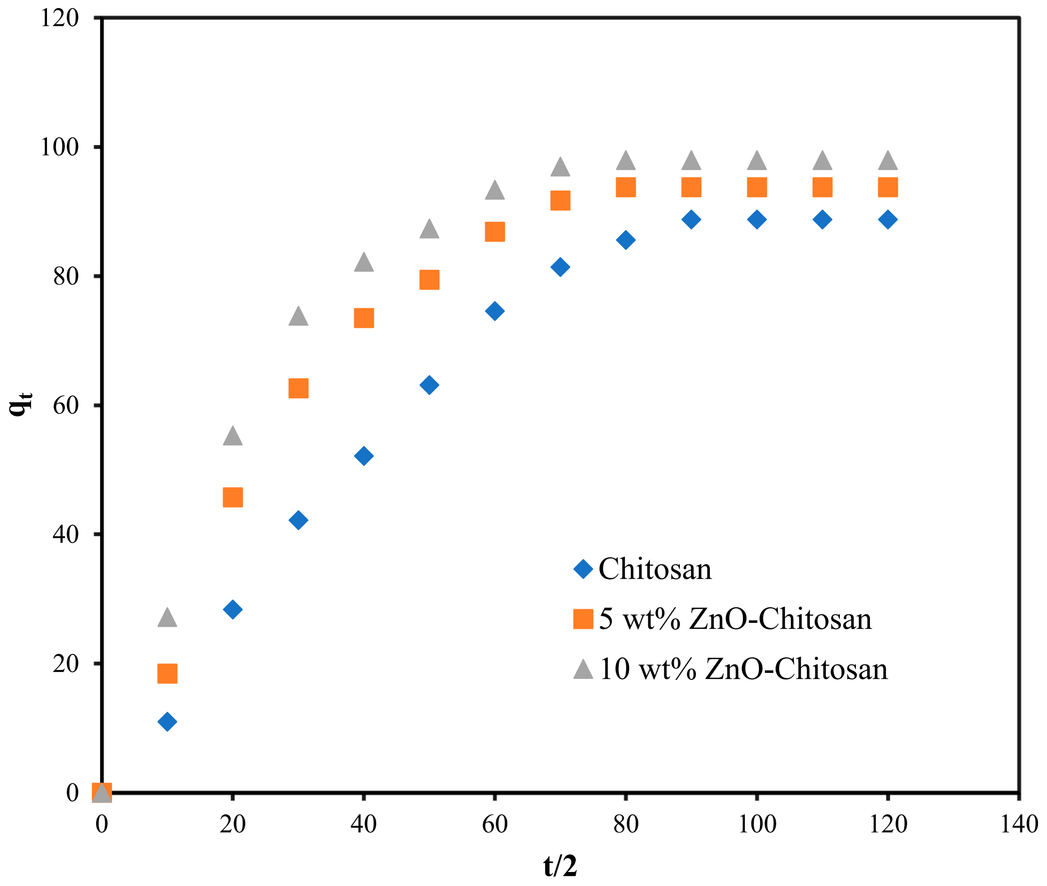

Figure S3. Intraparticle adsorption model plot for methylene blue adsorption onto chitosan and the nanocomposites.

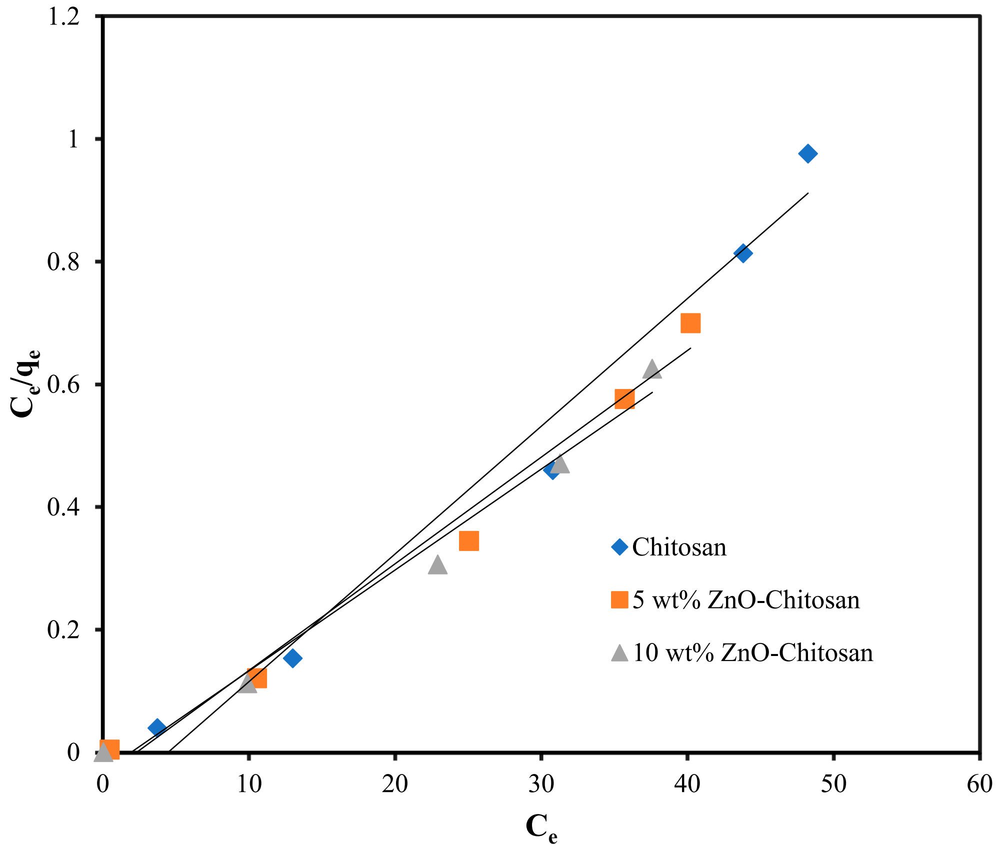

Figure S4. Langmuir isotherm model plot for methylene blue adsorption onto chitosan and the nanocomposites.

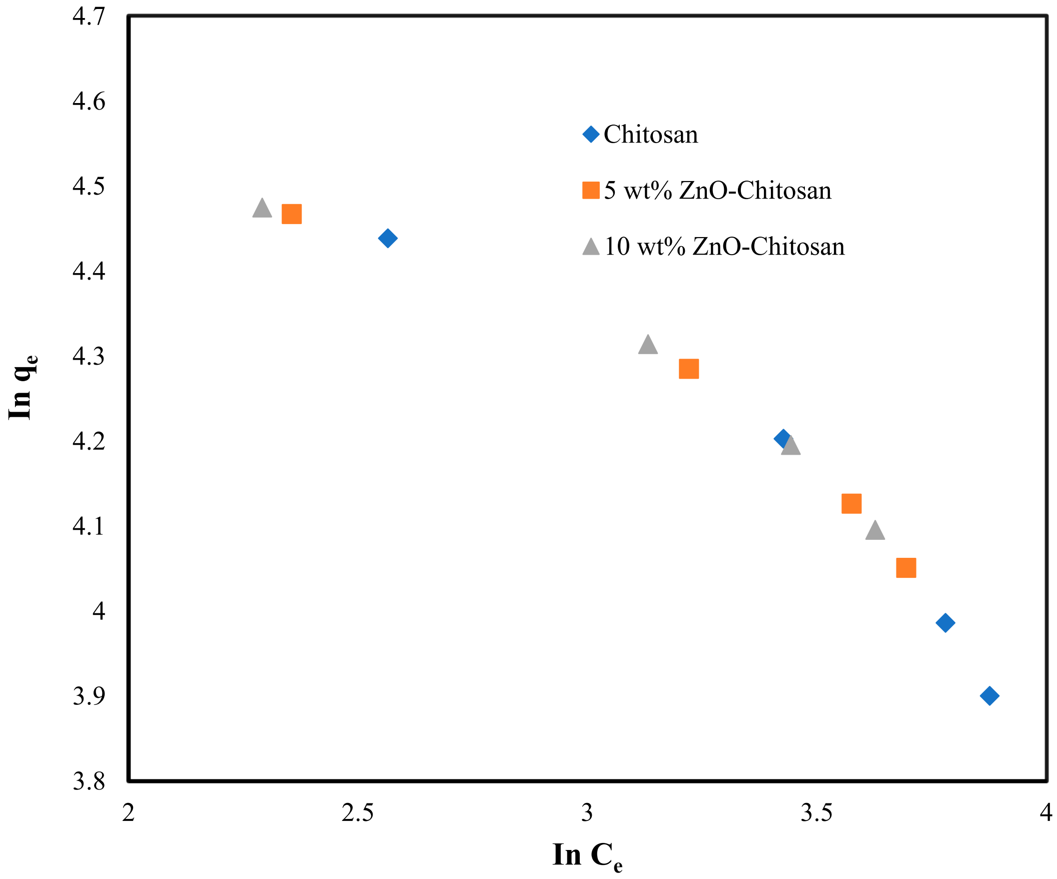

Figure S5. Freundlich isotherm model plot for methylene blue adsorption onto chitosan and the nanocomposites.

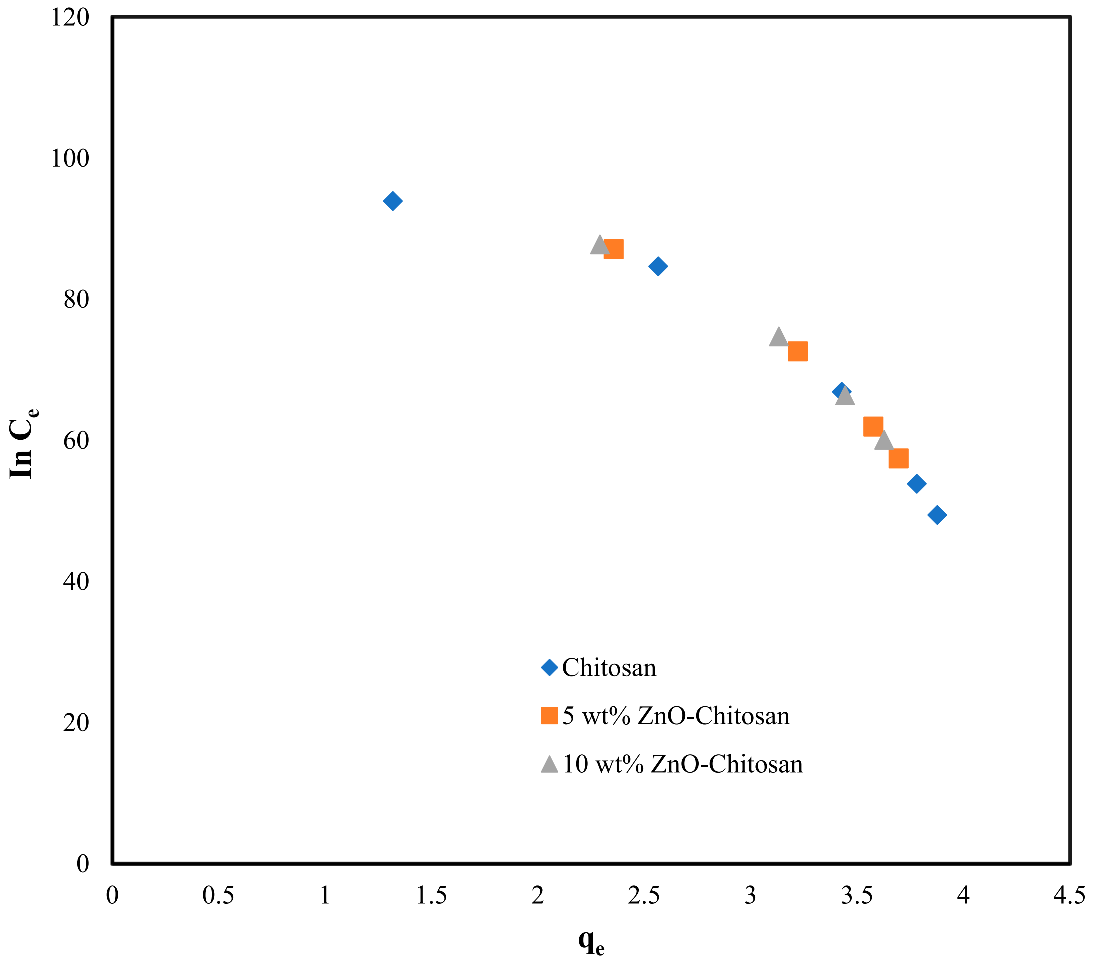

Figure S6. Temkin isotherm model plot for methylene blue adsorption onto chitosan and the nanocomposites.
